# Supplementary material for: Quantitative Real-Time PCR Based on SYBR Green Technology for the Identification of Philaenus italosignus Drosopoulos & Remane (Hemiptera Aphrophoridae)
Source: Plants (Basel). 2022 Nov 30;11(23):3314. doi: 10.3390/plants11233314 (PMC9741283; doi:10.3390/plants11233314)
Supplement: Supplementary file 1 [file plants-11-03314-s001.zip › plants-2028809-supplementary.pdf]

Figure S1. Complete alignment of Spit-Uni1/2 products: nine EF1-α alleles cloned from two *Philaenus italosignus* (Pi1 and Pi2) and two *P. spumarius* (Ps3 and Ps4) specimens collected during this study in Tuscany(Italy). The homologous sequences of the two alleles of P. spumarius PSPU_08 (GCA_018207615.1)and of the only allele of *P. italosignus* (JF309079) currently available in the Genbank database, were included for comparative purposes as PsRef1, PsRef2 and PiRef1, respectively. The exon region of the EF1-α alleles is underlined meanwhile, within the intron region, the positions of the *P. italosignus* specific qPCR assay primers, Pital_348F and Pital_465R, are highlighted in black and gray, respectively. The "*" character indicates positions that are fully conserved.

```
PiRef-1       AAACGTACCATTGAGAAGTTCGAGAAGGAAGCCCAAGAAGTAAGTTGACTA-----TACT   55
Pi1-1         AAACGTACCATTGAGAAGTTCGAGAAGGAAGCCCAAGAAGTAAGTTGACTA-----AACT   55
Pi1-2         AAACGTACCATTGAGAAGTTCGAGAAGGAAGCCCAAGAAGTAAGTTGACTA-----AACT   55
Pi2-1         AAACGTACCATTGAGAAGTTCGAGAAGGAAGCCCAAGAAGTAAGTTGACTA-----TACT   55
Pi2-2         AAACGTACCATTGAGAAGTTCGAGAAGGAAGCCCAAGAAGTAAGTTGACTA-----AACT   55
PsRef-1       AAACGTACCATTGAGAAGTTCGAGAAGGAAGCCCAAGAAGTAAGTTGACTA-----TACT   55
PsRef-2       AAACGTACCATTGAGAAGTTCGAGAAGGAAGCCCAAGAAGTAAGTTGACTA-----TACT   55
Ps3-1         AAACGTACCATTGAGAAGTTCGAGAAGGAAGCCCGAGAAGTAAGTTGACTATAGCCTACT   60
Ps3-2         AAACGTACCATTGAGAAGTTCGAGAAGGAAGCCCAAGAAGTAAGTTGACTATAGCCTACT   60
Ps4-1         AAACGTACCATTGAGAAGTTCGAGAAGGAAGCCCAAGAAGTAAGTTGACTA-----TACT   55
Ps4-2         AAACGTACCATTGAGAAGTTCGAGAAGGAAGCCCAAGAAGTAAGTTGACTA-----TACT   55
Ps4-3         AAACGTACCATTGAGAAGTTCGAGAAGGAAGCCCAAGAAGTAAGTTGACTA-----TACT   55
              ********************************** ****************      ***

PiRef-1       AGCAGAATAAAATTTGAATCAACTCCTATAAAAGCGAGTAACATATAGG---CGTAATAT   112
Pi1-1         AGCGGAATAAAATTTGAATCAACTCCTATAAAAGCGAGTAACATATATAGG-CCTAATAT   114
Pi1-2         AGCGGAATAAAATTTGAATCAACTCCTATAAAAACGAGTAACATATATAGG-CCTAATAT   114
Pi2-1         AGCGGAATAAAATTTGAATCAACTCCTATAAAAGCGAGTAACATATAGG---CGTAATAT   112
Pi2-2         AGCGGAATAAAATTTGAATCAACTCCTATAAAAGCGAGTAACATATATAGG-CCTAATAT   114
PsRef-1       AGCGGAATAAAATTTAAATGAACTCCTATAAAAACGAGTAACATA-------TATAATAT   108
PsRef-2       AGCGGAATAAAATTTAAATGAACTCCTATAAAAACGAGTAACATA-------TATAATAT   108
Ps3-1         AGCGGAATAAAATTTAAATCAACTCCTATAAAAACGAGTAACATA------------TAT   108
Ps3-2         AGCGGAATAAAATTTAAATCAACTCCTATAAAAACGAGTAACATA------------TAT   108
Ps4-1         AGCGGAATAAAATTTAAATCAACTCCTATAAAAGCGAGTAACATA-------TATTATAT   108
Ps4-2         AGCGGAATAAAATTTAAATGAACTCCTATAAAAACGAGTAACATATATAGCCTATAATAT   115
Ps4-3         AGCGGAATAAAATTTAAATGAACTCCTATAAAAACGAGTAACATATATAGCCTATAATAT   115
              *** *********** *** ************* ***********            ***

PiRef-1       AATAGCTGATTTCACCCATCATCTGTAATACATAAACATAGCCTAACTCGTGTAGGTTGT   172
Pi1-1         AACAGCTGATTTCACCCATCATCTGTAATACATAAACATAGCCTAACCCGTGTAGGTTGT   174
Pi1-2         AACAGCTGATTTCACCCATCATCTGTAATACATAAACATAGCCTAACCCGTGTAGGTTGT   174
Pi2-1         AATAGCTGATTTCACCCATCATCTGTAATACATAAACATAGCCTAACTCGTGTAGGTTGT   172
Pi2-2         AACAGCTGATTTCACCCATCATCTGTAATACATAAACATAGCCTAACCCGTGTAGGTTGT   174
PsRef-1       AATAGCTGATTTCACCAATCATCTGTAATACATAAACATAGCCTAACCCGTGTAGGTTGT   168
PsRef-2       AATAGCTGATTTCACCAATCATCTGTAATACATAAACATAGCCTAACCCGTGTAGGTTGT   168
Ps3-1         ATTAGCTGATTTCACCAATCATCTGTAATACATAAACATAGCCTAACCCGTGTAGGTTGG   168
Ps3-2         AATAGCTGATTTCACCAATCATCTGTAATACATAAACATAGCCTAACCCGTGTAGGTTGG   168
Ps4-1         AATAGCTGATTTCACCAATCATCTGTAATACATAAACATAGCCTAACCCGTGTAGGTTGT   168
Ps4-2         AATAGCTGATTTCACCAATCATCTGTAATACATAAACATAGCCTAACCCGTGTAGGTTGT   175
Ps4-3         AATAGCTGATTTCACCAATCATCTGTAATACATAAACATAGCCTAACCCGTGTAGGTTGT   175
              *  ************* ****************************** *********** 

PiRef-1       AGTTCGGGGTTATGTTTACAACTTCAATCGTTATAATTTCTTGTGAAGTAGATTTTTTTA   232
Pi1-1         AGTTCGGGGTTATGTTTACAACTTCAATCGTTATAATTTCTTGTGAAGTAGA-TTTTTTA   233
Pi1-2         AGTTCGGGGTTATGTTTACAACTTCAATCGTTATAATTTCTTGTGAAGTAGA-TTTTTTA   233
Pi2-1         AGTTCGGGGTTATGTTTACAACTTCAATCGTTATAATTTCTTGTGAAGTAGATTTTTTTA   232
Pi2-2         AGTTCGGGGTTATGTTTACAACTTCAATCGTTATAATTTCTTGTGAAGTAGA-TTTTTTA   233
PsRef-1       AGTTTGGGGTTATGTTTACAACTTCA------------------------------TTTT   198
PsRef-2       AGTTTGGGGTTATGTTTACAACTTCA------------------------------TTTT   198
Ps3-1         AGTTAGGGGTTATGTTTACAACTTCAATCGTTATAAATTCTTGTAAAGTTGA--TTTTTT   226
Ps3-2         AGTTAGGGGTTATGTTTACAACTTCAATCGTTATAATTTCTTGTAAAGTTGA--TTTTTT   226
Ps4-1         AGTTTGGGGTTATGTTTACAACTTCAATCGTTATAATTTCTTGTGAAGTTGA--TTTTTT   226
```

```
Ps4-2          AGTTTGGGGTTATGTTTACAACTTCAATCGTTATAATTTCTTGTGAAGTTGA-TTTTTTT   234
Ps4-3          AGTTTGGGGTTATGTTTACAACTTCAATCGTTATAATTTCTTGTGAAGTTGA-TTTTTTT   234
               **** *********************                              ***

PiRef-1        AAATTTGAGTTACAGAACTAATGTATGCTATAATATGACGTTATATTTGC-TTTGAGGTT   291
Pi1-1          AAATTTGAGTTACAGAACTAATGTATGCTATAATATGACGTTATATTTGC-TTTGAGGTT   292
Pi1-2          AAATTTGAGTTACAGAACTAATGTATGCTATAATATGACGTTATATTTGC-TTTGAGGTT   292
Pi2-1          AAATTTGAGTTACAGAACTAATGTATGCTATAATATGACGTTATATTTGC-TTTGAGGTT   291
Pi2-2          AAATTTGAGTTACAGAACTAATGTATGCTATAATATGACGTTATATTTGC-TTTGAGGTT   292
PsRef-1        TAATTTGAGTTAAAGAACTAATATATGCTATAATATGACGTTATATTTGCTTTTGAGGTT   258
PsRef-2        TAATTTGGGTTAAAGAACTAATATATGCTATAATATGACGTTATATTTGCTTTTGAGGTT   258
Ps3-1          TAATTTGAGTTACAGAACTAATATATGCTATAATATGACGTTATATTTGC-TTTAAGGTT   285
Ps3-2          TAATTTGAGTTACAGAACTAATATATGCTATAATATGACGTTATATTTGC-TTTAAGGTT   285
Ps4-1          TAATTTGAGTTAAAGAACTAATATATGCTATAATATGACGTTATATTTGCTTTTGAGGTT   286
Ps4-2          TAATTTGAGTTACAGAACTAATATATGCTATAATATGATGTTATATTTGC-TTTGAGGTT   293
Ps4-3          TAATTTGAGTTACAGAACTAATATATGCTATAATATGATGTTATATTTGC-TTTGAGGTT   293
                ****** **** ********* *************** *********** *** *****

PiRef-1        ATAAACTGTAGGCCTATGTAA-----------------TCA-TATATTTATTCAAAATTTA   334
Pi1-1          ATAAACTGTAGGCCTATGTAA-----------------TCAATATATTTATTCAAAATTTA   336
Pi1-2          ATAAACTGTAGGCCTATGTAA-----------------TCAATATATTTATTCAAAATTTA   336
Pi2-1          ATAAACTGTAGGCCTATGTAA-----------------TCAATATATTTATTCAAAATTTA   335
Pi2-2          ATAAACTGTAGGCCTATGTAA-----------------TCAATATATTTATTCAAAATTTA   336
PsRef-1        ATAAACTGTAGGCCTATGTAAATACAACCTTTTATCATCAATATATTTATTTAAAATTTA   318
PsRef-2        ATAAACTGTAGGCCTATGTAAATACAACCTTTTATCATCAATATATTTATTTAAAATTTA   318
Ps3-1          ATAAACTGTAGGCCTATGTAAATACAACCTTTTATCATGAATATATTTATTTAAAATTTA   345
Ps3-2          ATAAACTGTAGGCCTATGTAAATACAACCTTTTATCATGAATATATTTATTTAAAATTTA   345
Ps4-1          ATAAACTGTAGGCCTATGTAAATACAACCTTTTATCGTCAATATATTTATTTAAAATTTA   346
Ps4-2          ATAAACTGTAGGCCTATGTAAATACAATCTTTTATCATCAATATATTTATTTAAAATTTA   353
Ps4-3          ATAAACTGTAGGCCTATGTAAATACAATCTTTTATCATCAATATATTTATTTAAAATTTA   353
               *********************                   *********** ********

PiRef-1        AAGA-GATCTATTCGTTTAACTTCGGAATTATTCATAGCCCTATATATTCGTAAAATAAT   393
Pi1-1          AAGA-GATCTATTCGTTTAACTTCGGAATTATTCGTAGCCCTATATCTTCGTAAAATAAT   395
Pi1-2          AAGA-GATCTATTCGTTTAACTTCGGAATTATTCGTAGCCCTATATCTTCGTAAAATAAT   395
Pi2-1          AAGA-GATCTATTCGTTTAACTTCGGAATTATTCATAGCCCTATATATTCGTAAAATAAT   394
Pi2-2          AAGA-GATCTATTCGTTTAACTTCGGAATTATTCGTAGCCCTATATCTTCGTAAAATAAT   395
PsRef-1        AAGCTGATCTATTCGTTTAGCTTTGGAAACATTCGTAGCCCTATATCTTCGTAAAATAAT   378
PsRef-2        AAGCTGATCTATTCGTTTAACTTTGGAAACATTCGTAGCCCTATATCTTCGTAAAATAAT   378
Ps3-1          AAGCTAATCTATTCGTTTAACTT-GGAAACATTCGTAGCCCTATATCTTCGTAAAATAAT   404
Ps3-2          AAGCTAATCTATTCGTTTAACTT-GGAAACATTCGTAGCCCTATATCTTCGTAAAATAAT   404
Ps4-1          AAGCTGATCTATTCGTTTAACTTCGGAAACATTCGTAGCCCTATATCTTCGTAAAATAAT   406
Ps4-2          AAGCTGATCTATTCGTTTAACTTCGGAAACATTCGTAGCCCTATATCTTCGTAAAATAAT   413
Ps4-3          AAGCTGATCTATTCGTTTAACTTCGGAAACATTCGTAGCCCTATATCTTCGTAAAATAAT   413
               ***   ************* *** ****  **** *********** *************

PiRef-1        TACTATTTCATTATTAATTTCTCCATTATAGCCTAATATTAATAACTTCCAAATCATTTG   453
Pi1-1          TAATATTTCATTATTAATTTCTCCATTATAGAATA--ATTAATAACTTCCAAATTATTTG   453
Pi1-2          TAATATTTCATTATTAATTTCTCCATTATAGAATA--ATTAATAACTTCCAAATTATTTG   453
Pi2-1          TACTATTTCATTATTAATTTCTCCATTATAGCCTAATATTAATAACTTCCAAATCATTTG   454
Pi2-2          TAATATTTCATTATTAATTTCTCCATTATAGAATA--ATTAATAACTTCCAAATTATTTG   453
PsRef-1        TAATATTTCATTAGTCATTTCTCCATTATAGCCTAATATTAATAACTTCCAAATCATTTG   438
PsRef-2        TAATATTTCATTAGTCATTTCTCCATTATAGCCTAATATTAATAACTTCCAAATCATTTG   438
Ps3-1          TAATATTTCATTAGTCATTTCTCCATTATAGCCTAATATTAATAACTTCCAAATCATTTG   464
Ps3-2          TAATATTTCATTAGTCATTTCTCCATTATAGCCTAATATTAATAACTTCCAAATCATTCG   464
Ps4-1          TAATATTTCATTAGTCATTTCTCCATTATAGCCTAATATTAATAACTTCCAAATCATTTG   466
Ps4-2          TAATATTTCTTTAGTCATTTCTCCATTATAGCCTAATATTAATAACTTCCAAATCATTTG   473
Ps4-3          TAATATTTCTTTAGTCATTTCTCCATTATAGCCTAATATTAATAACTTCCAAATCATTTG   473
               ** ****** *** * ***************  **  ***************** *** *

PiRef-1        ATCTATCAAAATATGAAAT-AAAAGCAATTAAAAGATAGTATTTTTATCACATAATAACC   512
Pi1-1          ATCTATCAAAATATGAAAT-AAAAGCAATTAAAAGATAGTATTTTTATCACGTAATAACC   512
Pi1-2          ATCTATCAAAATATGAAAT-AAAAGCAATTAAAAGATAGTATTTTTATCACGTAATAACC   512
```

```
Pi2-1          ATCTATCAAAATATGAAAT-AAAAGCAATTAAAAGATAGTATTTTTATCACATAATAACC   513
Pi2-2          ATCTATCAAAATATGAAAT-AAAAGCAATTAAAAGATAGTATTTTTATCACGTAATAACC   512
PsRef-1        ATCTATC--AATATGAAAT-AAAAGCAATTAAAAGGTAGTATTTTTATCGCATAATAACC   495
PsRef-2        ATCTATC--AATATGAAAT-AAAAGCAATTAAAAGGTAGTATTTTTATCGCATAATAACC   495
Ps3-1          ATCTATC--AATATGAAATAAAAAACAATTAAAAGATAGTATTTTTATCGCACAATAACC   522
Ps3-2          ATCTATC--AATATGAAATAAAAAACAATTAAAAGATAGTATTTTTATCGCACAATAACC   522
Ps4-1          ATCTATC--AATATGAAAT-AAAAGCAATTAAAAGATAGTATTTTTATCGCATAATAACC   523
Ps4-2          ATCTATC--AATATGAAAT-AAAAGCAATTAAAAGATAGTATTTTTATCGCATAATAACC   530
Ps4-3          ATCTATC--AATATGAAAT-AAAAGCAATTAAAAGGTAGTATTTTTATTGCGTAATAACC   530
               *******  ********** **** ********** ************  *  *******

PiRef-1        TATGTAAATATAAAAGTTCAGCAAATAGGCCTACAGTTTTATAATCGTTATTACTGTACA   572
Pi1-1          TATGTAAATATAAAAGTTCAGCAAATAGGCCTACAGTTTTATAATCGTTATTACTGTACA   572
Pi1-2          TATGTAAATATAAAAGTTCAGCAAATAGGCCTACAGTTTTATAATCGTTATTACTGTACA   572
Pi2-1          TATGTAAATATAAAAGTTCAGCAAATAGGCCTACAGTTTTATAATCGTTATTACTGTACA   573
Pi2-2          TATGTAAATATAAAAGTTCAGCAAATAGGCCTACAGTTTTATAATCGTTATTACTGTACA   572
PsRef-1        TATGTAAATATAAATGTTCAGCAAATAGGCCTACAGTTTTATAATCGTTAT---------   546
PsRef-2        TATGTAAATATAAATGTTCAGCAAATAGGCCTACAGTTTTATAATCGTTAT---------   546
Ps3-1          TATGTAAATATAAATGTTCAGCAAATAGGCCTACAGTTTTATAATCGTTAT---------   573
Ps3-2          TATGTAAATATAAATGTTCAGCAAATAGGCCTACAGTTTTATAATCGTTAT---------   573
Ps4-1          TATGTAAATATAAATGTTCAGCAAATAGGCCTACAGTTTTATAATCGTTAT---------   574
Ps4-2          TATGTAAATATAAATGTTCAGCAAATAGGCCTACAGTTTTATAATCGTTAT---------   581
Ps4-3          TATGTAAATATAAATGTTCAGCAAATAGGCCTACAGTTTTATAATCGTTAT---------   581
               ************** ************************************

PiRef-1        CATTTATAGAGTTAAAAATCCCCGTCAGCATAGTTAGGTAGGAATAGATAAATATTCCTT   632
Pi1-1          CATTTATAGAGTTAAAAATCCCCGTCAGCATAGTTAGGTAGGAATAGATAAATATTCCTT   632
Pi1-2          CATTTATAGAGTTAAAAATCCCCGTCAGCATAGTTAGGTAGGAATAGATAAATATTCCTT   632
Pi2-1          CATTTATAGAGTTAAAAATCCCCGTCAGCATAGTTAGGTAGGAATAGATAAATATTCCTT   633
Pi2-2          CATTTATAGAGTTAAAAATCCCCGTCAGCATAGTTAGGTAGGAATAGATAAATATTCCTT   632
PsRef-1        ------TAGAGTTAAAAATCCCTGTCGGCGTAGTTAGGTAGGCCTAGATAAATATTCCTT   600
PsRef-2        ------TAGAGTTAAAAATCCCTGTCGGCGTAGTTAGGTAGGCCTAGATAAATATTCCTT   600
Ps3-1          ------TAGAGTTAAAAATCCCTGTCGGCATACTTAGGTAGGCCTAGATAAATATTCCTT   627
Ps3-2          ------TAGAGTTAAAAATCCCTGTCTGCATACTTAGGTAGGCCTAGATAAATATTCCTT   627
Ps4-1          ------TAGAGTTAAAAATCCCTGTCGGCA--------------TAGATAAATATTCCTT   614
Ps4-2          ------TAGAGTTAAAAATCCCTGTCGGCA--------------TAGATAAATATTCCTT   614
Ps4-3          ------TAGAGTTAAAAATCCCTGTCGGCGTAGTTAGGTAGGCCTAGATAAATATTCCTT   614
                     **************** *** **               ****************

PiRef-1        TAAGTTGTTTTTAAATTGTTTGTTTTTTAAATGATGTATTTCTGATTTGTATCTAATTAA   692
Pi1-1          TAAGTTGTTTTTAAATTGTTTGTTTTTTAAATGATGTATTTCTGATTTGTATCTAATTAA   692
Pi1-2          TAAGTTGTTTTTAAATTGTTTGTTTTTTAAATGATGTATTTCTGATTTGTATCTAATTAA   692
Pi2-1          TAAGTTGTTTTTAAATTGTTTGTTTTTTAAATGATGTATTTCTGATTTGTATCTAATTAA   693
Pi2-2          TAAGTTGTTTTTAAATTGTTTGTTTTTTAAATGATGTATTTCTGATTTGTATCTAATTAA   692
PsRef-1        TAAGTTGTTTTTAAATAGTTTGTTTTTTAAATGATGTATTTCTGATTTGTATCAAATTAA   660
PsRef-2        TAAGTTGTTTTTAAATAGTTTGTTTTTTAAATGATGTATTTCTGATTTGTATCAAATTAA   660
Ps3-1          TAAGTTGTTTTTAAATAGTTTGTTTTTTAAATGATGTATTTCTAATTTGTATCTAATTAA   687
Ps3-2          TAAGTTGTTTTTAAATAGTTTGTTTTTTAAATGATGTATTTCTGATTTGTATCTAATTAA   687
Ps4-1          TAAGTTGTTTTTAAATAGTTTGTTTTTTAAATGATGTATTTCTGATTTGTATCTAATTAA   674
Ps4-2          TAAGTTGTTTTTAAATAGTTTGTTTTTTAAATGATGTATTTCTGATTTGTATCTAATTAA   681
Ps4-3          TAAGTTGTTTTTAAATAGTTTGTTTTTTAAATGATGTATTTCTGATTTGTATCTAATTAA   695
               **************** ************************** ********* ******

PiRef-1        TCAGC   697
Pi1-1          TCAGC   697
Pi1-2          TCAGC   697
Pi2-1          TCAGC   698
Pi2-2          TCAGC   697
PsRef-1        TCAGC   665
PsRef-2        TCAGC   665
Ps3-1          TCAGC   692
Ps3-2          TCAGC   692
Ps4-1          TCAGC   679
```

```
Ps4-2          TCAGC   686
Ps4-3          TCAGC   700
               *****
```
